# A Lifespan Approach to Balance in Static and Dynamic Conditions: The Effect of Age on Balance Abilities

**DOI:** 10.3389/fneur.2022.801142

**Published:** 2022-02-21

**Authors:** Giorgia Marchesi, Alice De Luca, Valentina Squeri, Lorenzo De Michieli, Francesco Vallone, Alberto Pilotto, Alessandra Leo, Maura Casadio, Andrea Canessa

**Affiliations:** ^1^Spinal Cord Italian Lab (SCIL), Unità Spinale Unipolare, Santa Corona Hospital, Pietra Ligure, Italy; ^2^Department of Informatics, Bioengineering, Robotics, and Systems Engineering (DIBRIS), University of Genoa, Genoa, Italy; ^3^Movendo Technology srl, Genoa, Italy; ^4^Rehab Technologies, Italian Institute of Technology (IIT), Genoa, Italy; ^5^Department of Geriatric Care, Orthogeriatrics and Rehabilitation, Galliera Hospital, Genoa, Italy; ^6^Unità Spinale Unipolare, Ospedale Metropolitano Niguarda, Milan, Italy

**Keywords:** postural control, aging, static and dynamic assessment, standing balance, age-dependent changes, perturbations

## Abstract

Postural control is a complex sensorimotor skill that is fundamental to our daily life. The abilities to maintain and recover balance degrade with age. However, the time decay of balance performance with age is not well understood. In this study, we aim at quantifying the age-dependent changes in standing balance under static and dynamic conditions. We tested 272 healthy subjects with ages ranging from 20 to 90. Subjects maintained the upright posture while standing on the robotic platform hunova®. In the evaluation of static balance, subjects stood on the fixed platform both with eyes open (EO) and eyes closed (EC). In the dynamic condition, subjects stood with eyes open on the moving foot platform that provided three different perturbations: (i) an inclination proportional to the center of pressure displacements, (ii) a pre-defined predictable motion, and (iii) an unpredictable and unexpected tilt. During all these tests, hunova® measured the inclination of the platform and the displacement of the center of pressure, while the trunk movements were recorded with an accelerometer placed on the sternum. To quantify balance performance, we computed spatio-temporal parameters typically used in clinical environments from the acceleration measures: mean velocity, variability of trunk motion, and trunk sway area. All subjects successfully completed all the proposed exercises. Their motor performance in the dynamic balance tasks quadratically changed with age. Also, we found that the reliance on visual feedback is not age-dependent in static conditions. All subjects well-tolerated the proposed protocol independently of their age without experiencing fatigue as we chose the timing of the evaluations based on clinical needs and routines. Thus, this study is a starting point for the definition of robot-based assessment protocols aiming at detecting the onset of age-related standing balance deficits and allowing the planning of tailored rehabilitation protocols to prevent falls in older adults.

## Introduction

Postural control is a complex sensorimotor skill fundamental to maintain, achieve, or restore a state of balance during any daily life activity (1). The generation of effective and appropriate postural control commands requires the central nervous system to process sensory information and to integrate them with motor, premotor, and brainstem afferent signals (2). Aging alters postural control as it affects the central structures (3), the sensory system, both in terms of unimodal processing (4–6) and multisensory integration (7), and the motor functions, affecting both movement and force control (8).

However, while the decline due to age is well characterized when considering, for example, the number of mechanoreceptors (9) or the brain volume loss (10), there are limited studies that systematically evaluate the time decay of balance abilities with age.

Indeed, most studies investigating the effects of aging on postural control assessed the difference in performance between well age-separated groups of subjects, namely, young, middle aged, and old adults either in static (11–13) or in dynamic conditions (14–21). Unfortunately, all the above-mentioned studies include different age ranges, making their comparison difficult and introducing bias due to the specific selection of the age ranges for each group. This also prevents a clear identification of the onset and the deterioration rate of the balance abilities associated with aging.

Only recently, two studies looked at a wider age range compared to previous studies, trying to assess how different postural and walking parameters change over a continuum of age (22, 23). These two studies used a lifespan approach to provide a quantification of the decline of balance with age by combining linear regression and qualitative observations. Virmani et al. (23) studied to what extent age affects walking in different conditions (i.e., steady-state gait, dual-task walking, and tandem gait). Park et al. (22) analyzed the effect of age both on static balance and on gait, focusing on (a) balance during quiet stance, (b) anticipatory postural adjustments in gait initiation, and (c) dynamic balance during walking. However, the reactive components of postural control, such as postural adjustments to external perturbations or in the presence of unstable environmental conditions, are not studied despite these reactive components being fundamental to detect balance impairments and the risk of falling (2, 15, 24–27).

To the best of our knowledge, no studies evaluated the ability to maintain the upright posture both in static and dynamic conditions, focusing on the reactive components of postural control, as a function of age, considering a large cohort of subjects and spanning an interval of 70 years, i.e., from 20 to 90 years of age.

This study aims at filling this gap by describing the deterioration of balance performance in adulthood by considering the reactive components of balance, and has a two-fold purpose:

- Describe the deterioration of balance abilities as a mathematical function depending on age in both static and dynamic conditions;
- Determine the potential of different types of perturbations on probing balance abilities.

Also, in this study, we used a robotic platform that allowed us to investigate the reactive postural components of balance in a reliable, repeatable, and well-controlled manner. Importantly, the assessment protocol proposed in this work is designed for clinical evaluation. Thus, our testing conditions are intended to be quick and easy to perform. First, we assessed postural control in static condition with both eyes open (EO) and eyes closed (EC). We specifically investigate the role of visual feedback and the interplay between vision and aging while maintaining standing balance. Indeed, this is still a debated issue as some research (12, 28, 29) demonstrated that old adults rely more on vision, while others (20, 30) concluded that the rate of change due to EC is independent of age. Then, to test the reactive components of balance, we assessed postural control in three different dynamic conditions (2): (i) the perturbations depended on the subject himself and were proportional to the oscillation of the subject, (ii–iii) the perturbations were imposed by the robotic device and independent from the subjects. Specifically, those were continuous and predictable in (ii) and unpredictable and unexpected in (iii). In these dynamic tasks, which are more challenging than the static tasks, we expect to have a better-defined relationship between balance performance and age and/or a higher decay of the measured abilities.

## Materials and Methods

### Subjects

**Figure 1:**
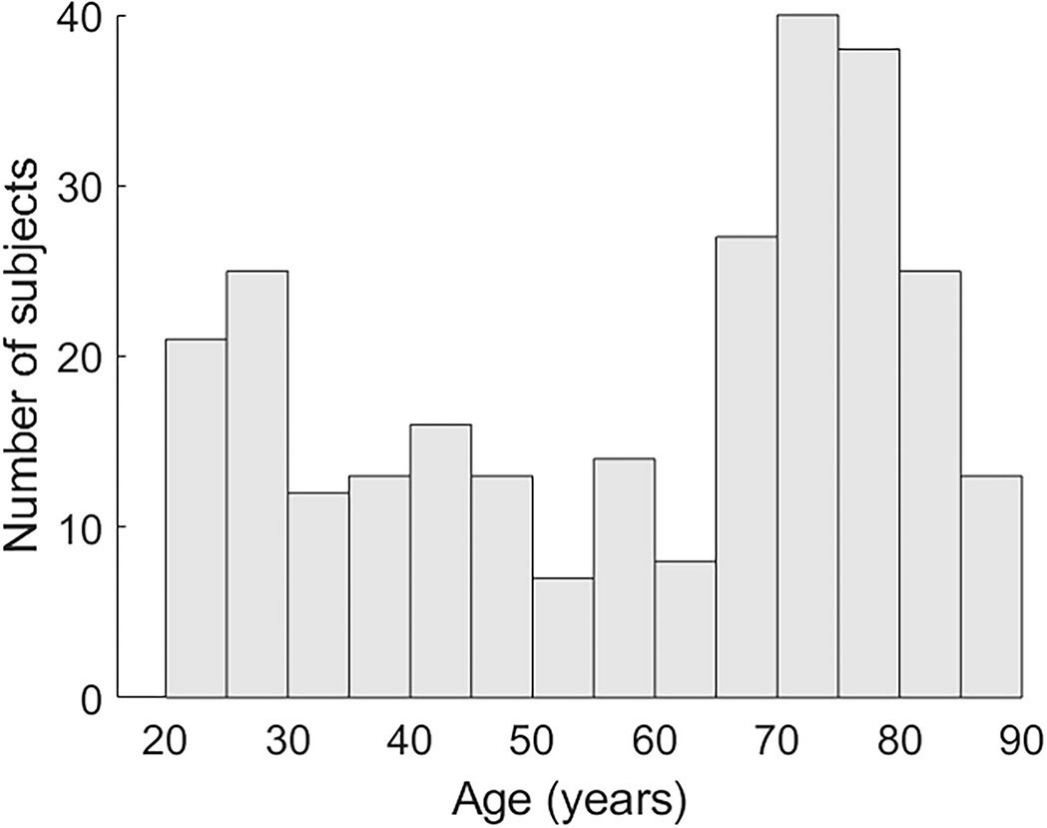
Age distribution of subjects.

A total of 272 healthy subjects (48 participants 19–30 years, 25 participants 31–40. 28 participants 41–50. 21 participants 51–60. 39 participants 61–70. 80 participants 71–80. 31 participants 81–90; see Figure 1 for the age distribution) participated in this study and matched the following criteria:

- age ranging from 19 to 90 years;
- absence of any neurological disorders (from the anamnesis) and/or moderate-severe cognitive impairment [subjects with more than 4/10 wrong answers to the Short Portable Mental Status Questionnaire (31) were excluded from the study];
- absence of any other condition that could affect balance;
- ability to stand and walk independently without assistive aids;
- absence of speech and/or aphasia disorders;
- absence of severe heart disease or respiratory failure.

Also, subjects that had a bone fracture in the 6 months (12 months in case of femoral fracture) prior to the evaluation were excluded. Participants were enrolled by the Department of Geriatric Care, Orthogeriatrics and Rehabilitation of Galliera Hospital (Genoa, Italy) in collaboration with the University of Genoa and the Italian Institute of Technology.

The study procedures conformed to the Declaration of Helsinki and were approved by the local ethical committees [Comitato Etico DIBRIS, reference number: CE DIBRIS: 012/2020 and Comitato Etico Regionale (CER) Liguria, reference number: 169REG2016]. All subjects included in the study signed a consent form that conforms to these guidelines and approved to publish individual data.

### Robotic Device

All subjects were tested using the medical robotic device hunova® from Movendo Technology srl, already described in previous studies (32, 33). Briefly, it has two electromechanical platforms: one under the feet and one under the seat (not used here) with two rotational degrees of freedom as described in (34). Behind each platform, a six-axis force-torque sensor allows the estimation of the center of pressure, while an optical incremental encoder allows the measurement of the inclination of the platforms. The device integrates an Inertial Measurement Unit (IMU) synchronized by software with the device. The IMU sensor in this experiment was placed on the sternum of the user for monitoring trunk motion, as previously done in previous studies (33, 35).

### Robotic Exercises and Protocol

During all tests, participants stood on the platform while wearing the IMU sensor on the sternum. At the beginning of each test, they were positioned on the platform with the heels separated by about 2 cm, the feet abducted at 20 degrees, and the arms relaxed along the sides of the body.

Participants were requested to stand still, avoiding any significant motion in all tests independently of the state of the foot platform. Before starting the experiment, subjects underwent a familiarization phase, where they become acquainted with the device and the proposed exercises by experiencing the platform movements and trying each exercise until they felt comfortable.

**Figure 2:**
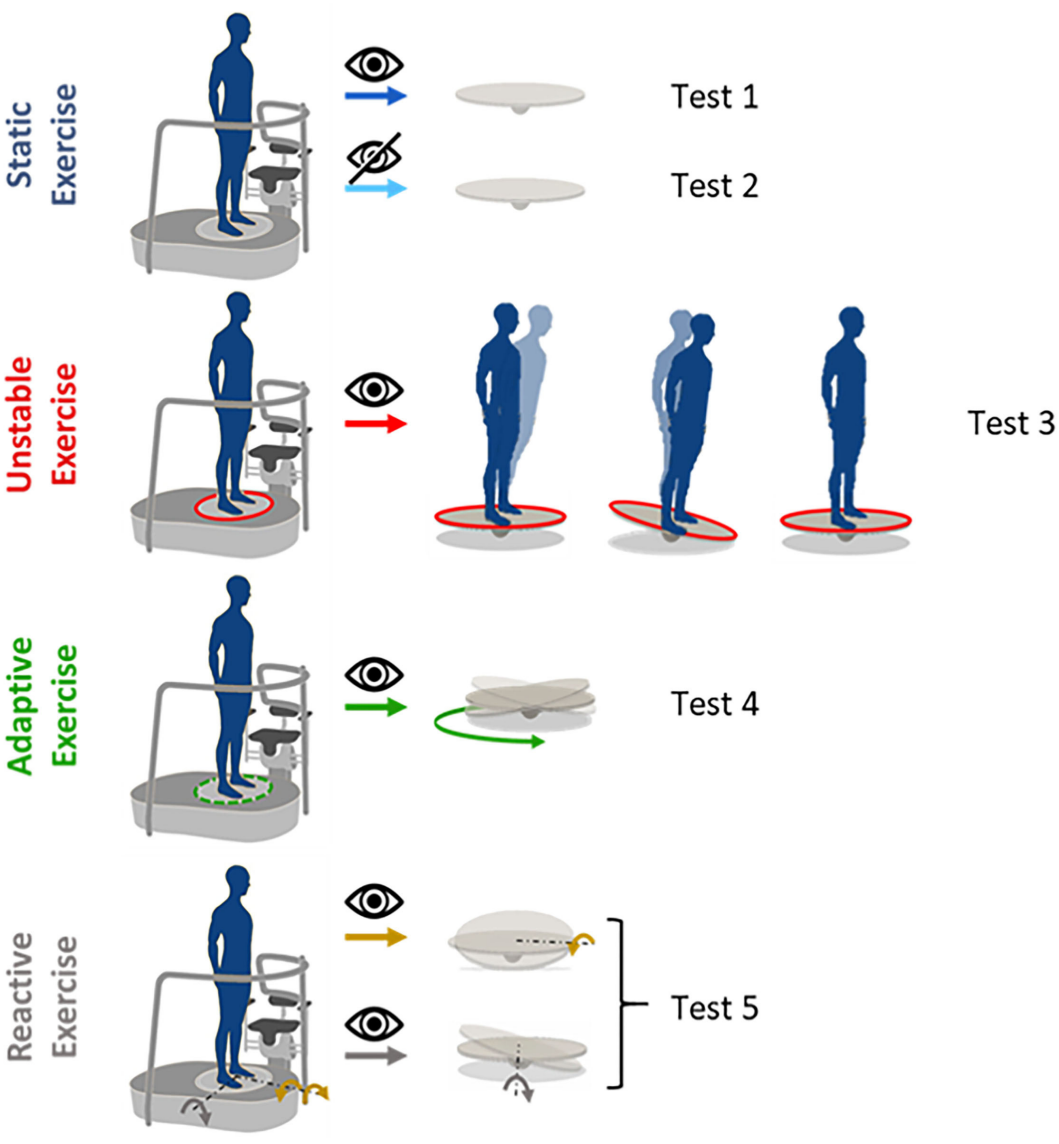
Proposed exercises: **static exercises** performed both with eyes open (test 1) and closed (test 2). The platform is kept fixed for the whole duration of the test; **unstable exercise** (test 3), the platform moves proportionally to the body's weight shift; **adaptive exercise** (test 4), the platform moves in a predictable and pre-programmed way on a circular trajectory; **reactive exercise with different perturbation directions** (test 5), the platform moves in a pre-programmed way, providing perturbations unpredictable for the users, tilting around the *x*-axis for the lateral perturbations (orange arrows, right-foot down and left-foot down) and around the *z*-axis, forward perturbation (gray arrow).

The protocol included five tests (see Figure 2), as follow:

*Test 1 and 2*. Static condition, i.e., the platform was kept fixed for the entire duration of the test. Participants had their eyes either open (EO—Test1) or closed (EC—Test2).
*Test 3-4-5*. Dynamic condition, i.e., the foot-platform was moving in three different ways described below. Participants always had their eyes open. Specifically:
*Test 3*. Subjects were asked to stand still on an unstable surface. The platform tilted in response to the weight shift of the subject. The platform responded as a plate on a pivot, with an additional low elastic rotatory force field that opposed to the movement induced by the subject weight shift and tended to restore the platform parallel to the floor.
*Test 4*. Subjects were asked to stand on the platform that was moving according to a preprogrammed and continuous circular trajectory (not influenced by the subject motion). The platform was tilting around the x and z axes, generating a circular trajectory given by the following equations [as previously described in a previous study (36)]:
  $$\begin{array}{l} \theta_{z}=A\text{ sin}\left(\pi \omega t\right)\\ \theta_{x}=A/2\text{ sin}\left(2\pi \omega t\right) \end{array}$$
  where θ_*z*_ and θ_*x*_ are the angular tilt around the medio-lateral (ML) and antero-posterior (AP) directions, respectively, and *A* is the maximum angular rotation and ω is the angular velocity. In our specific case, A = 6; ω = 0.15.
*Test 5*. Subjects were asked to stand on the platform while experiencing unpredictable perturbations. The platform tilted forward or laterally, along the z and x rotational axes, respectively. Thus, there were three possible perturbations: (i) “toes down” along the positive z-axis (i.e., forward perturbation), (ii) “right-foot down,” and (iii) “left-foot down” along the x-axis (i.e., rightward perturbation; leftward perturbation, respectively). In this exercise, the platform rotated following a Gaussian profile trajectory, as to respect the minimum jerk trajectory, with the peak of 5.5° at 330 ms after the perturbation onset (mean velocity ~16.5°/s). A total of nine perturbations, three for each perturbation direction, were presented in random order and with a jittered time interval between each one (4.7 + 0.6 s) to avoid anticipation or guessing.

In all the eyes open conditions, participants were asked to fix a single point on a wall 1 m away. In case participants did not finish one of the proposed exercises because they used the handles of hunova® to restore balance or opened their eyes in the EC test, they were requested to repeat the exercise after a break to prevent fatigue.

Notice that the coordinate reference system is the same commonly used for gait analysis with the positive *x*-, *y*-, and *z*-axes, respectively, pointing forward (AP direction), up, and right (ML direction), defining a right-handed system [for clarification, see (33)]. Positive rotations are counterclockwise about the axis of rotation. The center of the system is in the middle of the platform.

Based on *a priori* assumptions of clinicians, each performed exercise within the protocol (exception made by test 5) lasted 20 s. While this is not the classical balance test duration, clinicians believed this was sufficient to highlight the signs of decline in balance performance due to age that may qualify as risk biomarkers for preventable fall. This complied with the final aim of a clinically applicable and safe protocol that: (a) included different conditions but was also administrable in a reasonable time (i.e., around 10 min), (b) avoided the risk of falling in dynamic conditions which in their experience, could occur in some older participants under longer exposure.

For completeness, since in the literature a duration of 30 s is more common, in the Supplementary Figures S1, S2), data supporting the hypotheses that the posturographic analysis does not lead to different results when based on 20 and 30 s of recording are provided.

### Data Analysis

The trajectory followed by the platform in its motion and the signals from the IMU sensor were simultaneously recorded at a sampling frequency of 30 Hz and saved by hunova®. As previously explained in previous studies (33, 36), we used the IMU accelerations to evaluate balance performance. The acceleration measures from the IMU were firstly corrected to have them referred to a true horizontal-vertical Cartesian coordinate system (37) and then filtered with a 12 Hz cut-off low-pass Butterworth filter.

**Figure 3:**
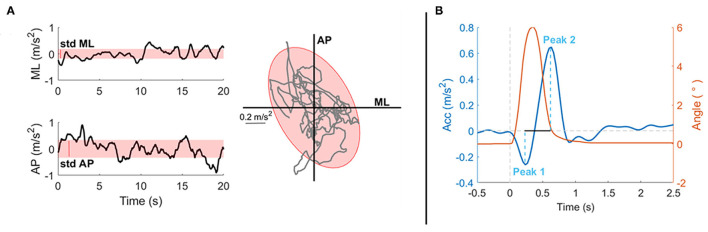
Experimental data from a representative subject to explain the parameters selected for the analysis of the proposed tasks. Parameters are based on readings of an Inertial Measurement Unit (IMU) placed on the sternum of the subject, i.e., parameters are extracted from the acceleration measures. **(A)** On the first column, the stabilograms (black line) and the variability (red shaded area, STD) of a 20-s exercise both in the ML (up) and AP (bottom) directions are shown. In the second column, the statokinesigram (gray line) together with the fitted confidence ellipse (red shaded area) representing the sway area are shown. Those parameters are computer for tests 1, 2, 3, and 4. **(B)** This panel shows the postural response after a perturbation of a representative subject for test 5: in red the perturbation trajectory, in blue the postural response along the perturbation's direction. Here, the peaks amplitudes are highlighted (light blue dashed line) together with the peak-to-peak time difference (black line).

From the trunk acceleration signals, different spatio-temporal parameters were computed (see below). For tests 1, 2, 3, and 4, the following parameters were extracted [as previously done in previous studies (33, 38) and as shown in Figure 3]:

- Mean velocity (MV, m/s): the mean value of the speed on the horizontal plane (39), i.e., the 2-norms of velocity along the *x*–*z*-axes, obtained by the integration of the corresponding components of the acceleration (40);
- Anterior-Posterior variability (STD AP, m/s^2^): standard deviation of the trunk acceleration along the AP direction (*z*-axis): the bigger this value, the more subjects moved in this direction;
- Medio-Lateral variability (STD ML, m/s^2^): standard deviation of the trunk acceleration along the ML direction (*x*-axis): the bigger this value, the more subjects moved in this direction;
- Sway area (SA, m^2^/s^4^): the area of the 95% confidence ellipse of the statokinesigram of the trunk accelerations in the horizontal plane (i.e., the surface that contains 95% probability of the individual points that make up the statokinesigram).

All the above parameters provide a comprehensive spatio-temporal description of the postural sway in the proposed tasks.

For test 5, data were segmented in 1.75 s lasting epochs, from −0.25 to 1.5 s after each perturbation onset. For each perturbation, the acceleration measure had the first peak in the direction of the perturbation and then, a rebound peak in the opposite direction. Hence, the following parameters were computed (Figure 3B):

- Peak_1_: amplitude of the first peak following the perturbation in the direction of the perturbation;
- Peak_2_: amplitude of the rebound peak in the opposite direction;
- P2P_amp_: peak-to-peak amplitude, amplitude distance between Peak_1_ and Peak_2_;
- P2P_time_: peak-to-peak time, time distance between Peak_1_ and Peak_2_.

The proposed measures considered both the first oscillations performed to counteract the platform inclination and the postural adjustment following the platform inclination, together with a comprehensive measure that considered both strategies. For each direction, the three repetitions were averaged, as we expected no adaptation after only three repetitions. Since no effect was found between left and right lateral perturbations (see Supplementary Figure S3), the two lateral perturbations were averaged together to distinguish only forward and lateral perturbations.

Each computed parameter in each exercise was described and modeled as a function of age. To perform a comparison between all the parameters, each parameter was normalized in a relatively normalized performance index *P*_*i*_, *with i* = 1, .. *N*, where *N* is the number of parameters computed in the analysis. To do this, each measured parameter *m*^*i*^ was subtracted and then divided by a reference value $m_{0}^{i}$, obtained by the average value of all the subjects with an age under 25:


$$\begin{array}{l} P_{i}=\;\frac{m^{i}-m_{0}^{i}}{m_{0}^{i}} \end{array}$$

Then, each performance index, *P*^*i*^ was modeled as a function of age (*y*) using a second-order polynomial fitting curve:


$$\begin{array}{l} P_{i}\left(y\right)=\;a_{i}\;{\left(\frac{y-y_{0}}{y_{0}}\;\right)}^{2} \end{array}$$

where *y*_0_ represented the reference age value that we consider equal to 25. This procedure kept the model simple and dependent only on one fitting parameter, *a*_*i*_, which represented the rate of changes in performance due to age. Higher *a*_*i*_ were related to faster changes in performance throughout the lifespan. In our case, as we expected a negative impact of age, higher *a*_*i*_ meant a greater balance deterioration.

### Statistical Analysis

Each performance index was described by a second-order polynomial function that we defined fitting our data. As this study aimed at evaluating the effect of age on different balance performances over a wide healthy population and the focus was on the average subjects' performance, not on individual subjects, we used a robust fitting method to reduce the effects of outliers (41). Specifically, robust fitting weighs the contribution of each single data point to the fitting curve with a weight ranging from 0 to 1, and we excluded data with weights lower than 0.1, considering them outliers.

For each parameter of each exercise, the fitting was completely characterized by *a*_*i*_, which is reported with its confidence interval (with 95% confidence bounds). To evaluate the goodness of fit, we computed both the coefficient of determination (*R*^2^) and the square root of the variance of the residuals (RMSE).

Also, we divided our population into two groups, considering subjects under 50 and over 50 as in a previous study (23) to: (a) make our study comparable with other works and with (22, 23) (i.e., the other two works that assessed balance abilities considering age as a continuum) which also split their population into groups; and (b) make sure of the significance of our mathematical function. To test the significance of our results, we then tested the differences in performance in these two age groups, running either an unpaired *t*-test or a Wilcoxon rank-sum test (42) depending on the results of the normality test [Anderson Darling test (43)]. Significance was set for all statistics at the family-wise error rate of α = 0.05. Finally, we confirmed the strength and validity of our results by computing and reporting (in the Supplementary Material) the power analysis related to this comparison. Given the effect size, the sample size, and α, we computed the power of our result.

## Results

All subjects successfully completed all the proposed exercises without experiencing fatigue.

**Figure 4:**
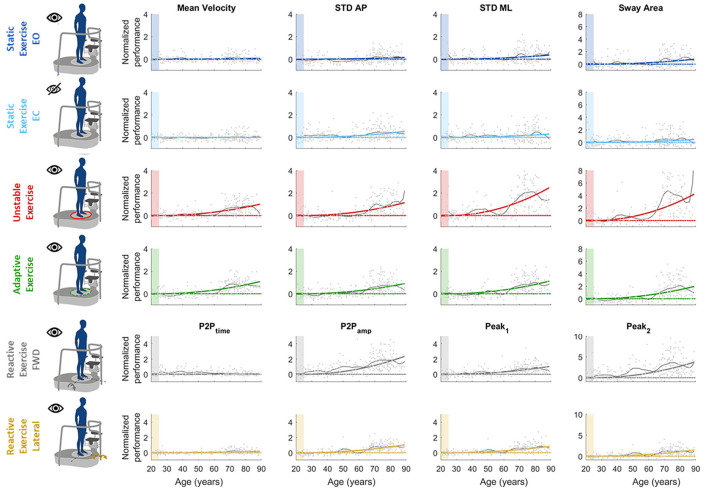
Computed parameters for all the performed tests. Each graph represents how each single parameter changes with age [*x-*axis: age (years), *y*-axis: normalized performance indexes]. In each graph, dots represent single subjects' performance; colored line is the parabolic fitted curve; black line represents the age by age mean curve; dashed color line is the reference performance (*y* = 0). The colored shaded patch highlights the reference age windows used for normalization (age between 20 and 24). Each row is relative to a different test: namely (from top to bottom) static EO, static EC, unstable, adaptive, reactive exercise (forward and lateral perturbation). Each column is relative to a specific computed parameter, namely, (from left to right) mean velocity (MV), STD AP, STD ML, sway area (SA, for test 1–4), and P2P_time_, P2P_amp_, Peak_1_, and Peak_2_, (for test 5).

We found that a quadratic function was suitable to describe the relationship between balance performance and age during most postural tasks, with a better fit for the dynamic conditions (see also Supplementary Material for comparison with different fitting functions). The fact that this function well describes the changes in balance abilities with age, considering the entire adult lifespan and without abrupt changes at a specific age, suggested that balance abilities have a continuous smooth degrade with age, with a higher decline later in life (i.e., at an older age), especially in dynamic conditions. Table 1 shows the fitting parameter, *a*_*i*_, which represents how fast performance changes due to age: higher values of *a*_*i*_ indicate faster changes, i.e., greater decline of balance ability throughout the adult life span. Tables 2, 3 show the coefficient of determination to describe the goodness of fit, *R*^2^, and the square root of the variance of the residuals, RMSE, both for each parameter in each exercise. The deterioration due to age of balance performance was highly dependent on the testing conditions, i.e., on the task (Figure 4; Table 1). Table 4 shows the results of the comparison between the performance of subjects under and over 50. These results are described in detail below.

**Table 1:** *a*_*i*_*-value*, reported with its confidence interval (with 95% confidence bounds).

	**MV**	**STD AP**	**STD ML**	**Area**
Static EO	0.012 (0.001–0.022)	0.024 (0.008–0.039)	0.055 (0.037–0.074)	0.121 (0.089–0.152)
Static EC	0.001 (0.000–0.002)	0.080 (0.061–0.099)	0.045 (0.025–0.064)	0.070 (0.038–0.102)
Unstable	0.154 (0.136–0.172)	0.174 (0.150–0.198)	0.377 (0.340–0.415)	0.643 (0.565–0.720)
Adaptive	0.164 (0.147–0.182)	0.138 (0.118–0.158)	0.171 (0.149–0.193)	0.301 (0.257–0.344)
	**P2P**_**time**_	**P2P**_**amp**_	**Peak**_**1**_	**Peak**_**2**_
Reactive, FWD	0.009 (0.000–0.017)	0.358 (0.324–0.392)	0.156 (0.138–0.174)	0.572 (0.497–0.646)
Reactive, lateral	0.031 (0.024–0.039)	0.157 (0.141–0.174)	0.127 (0.114–0.140)	0.225 (0.199–0.252)

*The a_i_ -value is the coefficient of the second-order curve that fits the postural parameter P_i_ expressed as a function of age. Higher values of a_i_ indicate faster changes, i.e., greater decline of balance ability throughout the adult life span. Each a_i_ -value is referred to a specific parameter and a specific exercise. Each row is referred to the exercise reported in the corresponding row of the first column, namely, static EO, static EC, unstable, adaptive, reactive FWD, and reactive lateral. In the upper part of the table, each column is referred to the parameter indicated in the first row: MV (mean velocity), STD AP (Antero-Posterior variability), STD ML (Medio-Lateral variability), and SA (sway area). In the lower part of the table, each column is referred to the parameter indicated in the seventh row, i.e. P2P_amp_ (peak-to-peak amplitude), P2P_time_ (time distance between Peak_1_ and Peak_2_), peak_1_ (amplitude of the first peak), and peak_2_ (amplitude of the second peak)*.

**Table 2:** Goodness of fit, R^2^, for the second order curve that fits a postural parameter P_i_, expressed as function of age.

	**MV**	**STD AP**	**STD ML**	**Area**
Static EO	0.161	0.039	0.110	0.265
Static EC	0.090	0.125	0.110	0.203
Unstable	0.328	0.393	0.445	0.616
Adaptive	0.393	0.358	0.350	0.456
	**P2P**_**time**_	**P2P**_**amp**_	**Peak**_**1**_	**Peak**_**2**_
Reactive, FWD	0.535	0.366	0.375	0.360
Reactive, lateral	0.326	0.424	0.539	0.371

*Higher values of R^2^ indicate better fit. Each R^2^ is referred to the fitting of a specific parameter in a specific exercise. Each row is referred to the exercise reported in the corresponding row of the first column, namely static EO, static EC, unstable, adaptive, reactive FWD, and reactive lateral. In the upper part of the table, each column is referred to the parameter indicated in the first row: MV (mean velocity), STD AP (Antero-Posterior variability), STD ML (Medio-Lateral variability), and SA (sway area). In the lower part of the table, each column is referred to the parameter indicated in the seventh row, i.e. P2P_amp_ (peak-to-peak amplitude), P2P_time_ (time distance between Peak_1_ and Peak_2_), peak_1_ (amplitude of the first peak), and peak_2_ (amplitude of the second peak)*.

**Table 3:** Square root of the variance of the residuals, RMSE.

	**MV**	**STD AP**	**STD ML**	**Area**
Static EO	0.271	0.406	0.467	0.801
Static EC	0.267	0.487	0.487	0.784
Unstable	0.461	0.584	0.900	1,711
Adaptive	0.450	0.503	0.563	1,052
	**P2P**_**time**_	**P2P**_**amp**_	**Peak**_**1**_	**Peak**_**2**_
Reactive, FWD	0.219	0.885	0.473	1,931
Reactive, lateral	0.199	0.436	0.338	0.684

*Specifically, each row is referred to the exercise reported in the corresponding row of the first column, namely static EO, static EC, unstable, adaptive, reactive FWD and reactive lateral. In the upper part of the table, each column is referred to the parameter indicated in the first row: MV (mean velocity), STD AP (Antero-Posterior variability), STD ML (Medio-Lateral variability) and SA (sway area). In the lower part of the table, each column is referred to the parameter indicated in the seventh row, i.e., P2P_amp_ (peak-to-peak amplitude), P2P_time_ (time distance between Peak_1_ and Peak_2_), peak_1_ (amplitude of the first peak), and peak_2_ (amplitude of the second peak)*.

**Table 4:** Mean ± std of each measured parameter *m*^*i*^ before normalization.

		**MV (m/s)**	**STD AP (m/s^**2**^)**	**STD ML (m/s^**2**^)**	**Area (m^**2**^/s^**4**^)**
Static EO	Under 50	0.270 ± 0.075	0.083 ± 0.030	0.043 ± 0.016	0.065 ± 0.040
	Over 50	0.279 ± 0.077	0.104 ± 0.040	0.050 ± 0.021	0.090 ± 0.055
Static EC	Under 50	0.280 ± 0.061	0.097 ± 0.034	0.053 ± 0.018	0.100 ± 0.058
	Over 50	0.320 ± 0.097	0.119 ± 0.047	0.060 ± 0.028	0.126 ± 0.084
Unstable	Under 50	0.446 ± 0.129	0.108 ± 0.049	0.077 ± 0.037	0.157 ± 0.143
	Over 50	0.670 ± 0.237	0.181 ± 0.079	0.158 ± 0.076	0.473 ± 0.332
Adaptive	Under 50	0.612 ± 0.192	0.159 ± 0.055	0.118 ± 0.040	0.351 ± 0.204
	Over 50	1,044 ± 0.340	0.255 ± 0.101	0.210 ± 0.086	0.903 ± 0.570
		**P2P**_**time**_ **(s)**	**P2P**_**amp**_ **(m/s**^**2**^**)**	**Peak**_**1**_ **(m/s**^**2**^**)**	**Peak**_**2**_ **(m/s**^**2**^**)**
Reactive, FWD	Under 50	0.409 ± 0.132	1,139 ± 0.461	0.739 ± 0.262	0.413 ± 0.283
	Over 50	0.365 ± 0.084	2,033 ± 0.893	1,111 ± 0.402	0.926 ± 0.612
Reactive, lateral	Under 50	0.307 ± 0.052	0.814 ± 0.226	0.360 ± 0.071	0.455 ± 0.188
	Over 50	0.324 ± 0.078	1,385 ± 0.462	0.531 ± 0.174	0.855 ± 0.370

*The colors are used to report statistical results of the comparison between subjects under 50 and subjects over 50: in red the values which resulted statistically significant (p < 0.001), in gray parameters with a non-statistically significant p, but with p ~ 0.1. Specifically, for each exercise, for each parameter, we reported mean ± std both for subjects under and over 50. Each row is referred to the exercise reported in the corresponding row of the first column, namely static EO, static EC, unstable, adaptive, reactive FWD, and reactive lateral. In the upper part of the table, each column is referred to the parameter indicated in the first row: MV (mean velocity), STD AP (Antero-Posterior variability), STD ML (Medio-Lateral variability), and SA (sway area). In the lower part of the table, each column is referred to the following parameters: P2P_amp_ (peak-to-peak amplitude), P2P_time_ (time distance between Peak_1_ and Peak_2_), peak_1_ (amplitude of the first peak), and peak_2_ (amplitude of the second peak)*.

### Static Tasks

In the static tasks, the deterioration of balance performance due to age was smaller and with a slower deterioration compared to all the dynamic tasks, i.e., overall aging had smaller effects on static than on dynamic performance. In the static test with EO, the age-dependent changes in the SA were due to the amplitude of the oscillations in the ML direction, while those in the AP changed with a slower rate (smaller *a-value* in the STD AP, as shown in Table 1). Also, the MV had negligible changes due to age (Table 1; Figure 4), as also confirmed by the comparison between under and over 50 (Table 4) which shows no statistical difference. In the EO condition, it is important to notice that all the parameters but the MV show a statistically significant difference between under and over 50 (Table 4).

Differently, in the static test with EC, the age-dependent changes of the SA were smaller compared to the EO (smaller *a-value*) as the relative difference between young and old adults is less marked as confirmed from the differences in performance of subjects under and over 50. Also, the variability of the oscillation in the mediolateral direction (STD ML) was equal in the EO and in the EC condition, i.e., the *a-value* defining the function that describes the decline with age of this parameter did not change depending on the availability of visual feedback. Instead, the oscillations in the anteroposterior direction (STD AP) changed depending on the EO–EC testing conditions. Specifically, the *a-value* for this parameter was higher in the EC condition (with also a statistical difference between under and over 50), indicating a greater change with respect to EO, i.e., the performance explained by this parameter degraded more in absence of visual feedback.

### Dynamic Tasks

In the unstable exercise, where the platform motion depended on the weight shift of the subject, and in the adaptive, where the subject needed to adapt to a continuous and predictable platform motion, the age-dependent changes were relevant for all parameters and statistically different when comparing under and over 50. Specifically, the unstable exercise had bigger changes (higher *a-value*) also associated with higher values of the goodness of fit. In addition, the SA, accounting for changes in both AP and ML directions, was the parameter that has the best fit with the parabolic curve for all the testing conditions.

Conversely, in the reactive exercise, the timing of the postural responses (P2P_time_) after a perturbation was not or was minimally affected by age (Tables 1, 4). Instead, P2P_amp_ had age-dependent changes marked more for perturbations in the forward direction. The difference was mainly due to the amplitude of the second peak (Peak_2_) which significantly changed with age and with a faster rate for perturbations in the forward directions. However, for the amplitude measures, the goodness of fit was always slightly better for the lateral than for the forward perturbations (Table 2).

### Performance of the Adults Under 25 Years of Age in the Different Testing Conditions

**Figure 5:**
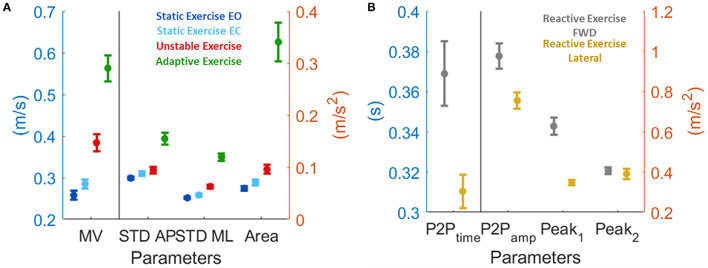
Motor performance of the subjects under 25 expressed as mean and standard deviation. This is the normalization factor we used before applying the fitting (see methods section for more details). **(A)** for test from 1 to 4, and **(B)** for test 5.

The performances of subjects under 25 have been considered as reference (i.e., normalization factor, as explained in the Method section). Subjects under 25 had a motor performance that depended on the task (Figure 5). All the parameters we selected had the same trend: lower values were typical of the easiest testing condition, i.e., the static with EO, and increase with the difficulties of the task following this order: static exercise with EC, unstable, and adaptive exercises. As for the reactive exercise, subjects under 25 had different results depending on the two perturbation directions: in the forward, both the first and the second peaks were bigger when compared to the lateral perturbations, along with the peak-to-peak timing. Indeed, the postural responses after an impulsive perturbation were longer when the perturbation is along the AP direction (i.e., forward perturbation).

## Discussion

In this study, we proposed a setup and a protocol to test balance abilities in different testing conditions, focusing on the reactive components of balance. The ability to maintain equilibrium when facing perturbations and environmental challenges is a fundamental component of balance that is necessary to avoid falls (2, 15, 24–27). Here, we considered the entire adult age span, and we highlighted that the decline of balance abilities with age could be described by a quadratic curve. Especially in the dynamic tests where the reactive component plays a major role, we observed an increase in the rate of decline with age, suggesting that a quadratic curve better describes than a linear fitting the decline with age. To comprehensively quantify the age-dependent changes in balance abilities, we also evaluated the influence of visual feedback while maintaining the standing posture in the static condition. We decided *a priori* to discard from this study the assessments in the dynamic condition in absence of visual feedback. This choice was motivated by the desire of the clinicians to define a safe protocol to test subjects without the risk of falling. Based on clinical practice, they judged the dynamic tasks with eyes closed associated with a high risk of falls, and they wanted *a priori* to exclude this condition from the protocol.

The effect of age on postural control was also clear from previous studies (11–18) that, differently from our approach, assessed balance differences dividing the population in few “age-groups,” e.g., considering young, middle age, and old subjects, with different definitions on the ranges across studies. Indeed, Allum et al. (19) assessed postural control after unexpected perturbations in the four directions including healthy subjects from 20 to 75 years of age, and they split the population into three groups: the first with age ranging between 20 and 34 years, the second 35–55, and the third 60–75. Liaw et al. (21) studied balance on a static platform with both eyes open and closed, dividing the population into three “age groups”: the first 18–39, the second 40–59, and the third 60–80 years old. Moreover, Freitas et al. (18) split the middle age group into two sub-groups and studied postural responses after forward perturbation in young adults (20–25), middle age 1 (40–45), middle age 2 (50–55), and old adults (60–65). Differently, Colledge et al. (13) divided the over 60 into two different groups: 60–70 and over 70. Moreover, the lack of a single definition for “young” and “old” led to different results. For this reason, here, we considered age as a continuous factor with no division in “age-groups.”

Before quantitatively assessing the age-dependent deterioration of balance abilities, we selected the best curve to fit our data. A previous study from Park et al. (22) studied the effect of age both on static balance and gait. They computed 37 different parameters and underlined three different typical trends depending on age: linear deterioration, decline after plateau, and no or minimal worsening. Differently, in our study, we found that there is a smooth deterioration of performance with age that could be described by a parabolic curve especially in dynamic conditions. This fitting allowed us to maintain a simple, i.e., depending on a single parameter, and easy-to-use model.

We found that the rapidity with which the performance changes depends on the selected postural parameter and the testing condition.

In the static condition, we compared performance with EO and EC. In literature, there is no unique opinion on the strategy subjects adopt to compensate for the absence of visual feedback. Sarabon et al. (30) concluded that older adults do not rely on vision more than young adults, while Benjuya et al. (29) highlighted the different strategies old and young adults adopt to compensate for the absence of visual feedback. From this study, the authors concluded that young adults compensate for the absence of vision with the use of other sensory information, while old adults stiff the ankles and co-contract agonist and antagonist leg muscles (29). Our results support the hypothesis of dependence on visual feedback of the deterioration of static balance performance with age. Indeed, we found bigger changes with age in the EC condition than in EO condition for the postural sway in the AP direction, i.e., the changes due to age were bigger in the EC condition. Instead, the age-dependent changes were not significantly different between the two feedback conditions for all the other parameters despite that subjects had a worse performance with eyes closed as expected, as shown by the normalization factor, i.e., the performance of subjects under 25 years of age. In summary, our results support the conclusion that older adults rely more on vision than younger adults in static standing balance tasks, and this is mainly observable in the AP postural sway.

As for the dynamic exercises, we included in our experiment three dynamic conditions to test different aspects related to reactive balance: the postural responses after unpredictable and unexpected external-perturbations, after predictable and continuous external-perturbations, and after auto-induced perturbations, i.e., the weight shift of the subject caused a tilt of an unstable platform. In all these exercises and in all the computed parameters, we found evident age-related changes. More precisely, among the dynamic tests, the unstable exercise was the most challenging one. Here, the small postural adjustments, if not optimally controlled, can cause auto-induced perturbations as each weight shift is transformed in a platform inclination. This exercise was the one that causes the biggest changes in performance with respect to young adults. Concerning the reactive exercise, the forward perturbation was the one that induced a bigger postural response with the biggest age effect.

Similar dynamic exercises are proposed in other studies (15, 18). However, several previous works mainly aimed at deeply understanding specific mechanisms underlying postural control and mainly focused on the comparison of performance between healthy subjects and people with well-known impairments. An example is a study on de-afferent subjects that clarified the role of sensory feedback (44–46). Alternatively, postural control was also described depending on its sensory processing and how it changed when the information of at least one of the sensory modalities (i.e., visual) was unavailable or modified (i.e., when we close our eyes). Other studies isolated single aspects of postural control, as defined in a previous study (2), investigating balance only under specific conditions as: (i) balance during quiet stance, (ii) reactive postural adjustments to external perturbations, (iii) anticipatory postural adjustments in preparation for voluntary movements, and (iv) dynamic balance during movements.

Here, we proposed a comprehensive, exhaustive, and short evaluation, suitable for assessment in clinical settings targeting different balance components, i.e., considering the role of visual feedback and specific aspects of the reactive postural control as defined in previous studies (2, 47), namely, the reactive postural adjustments to external perturbations, and dynamic balance during movements. All our metrics have been computed from the IMU placed on the sternum that provides reliable measures of balance abilities as demonstrated by Marchesi et al. (33), Mancini et al. (38, 48). In addition, in our exercises, we used a mobile feet force platform to provide different dynamic interactions in a controlled manner. The use of a robotic platform in our setup allowed us to expose subjects to different environmental conditions that can be repeatable and well-controlled. Indeed, robotic platforms are powerful tools offered to clinicians allowing for standardized assessments. This latter is a fundamental requirement when testing, as in our case, a large population to assess the decline of reactive balance abilities with age. Also, the use of robotic tools and platforms allows quantifying performance in an accurate and precise manner, reducing the subjective component added by the clinical test based on the evaluation of the operator.

In this work, we characterized how age affects balance describing the physiological changes of balance due to age. We concluded that, as expected, those changes are continuous. However, as balance degrades with age, strength and the ability to precisely control handgrip force are also well known to decrease with age. In addition, dynamic balance and handgrip strength seem to be correlated (49), and we could expect a correlation that is worth investigating in future studies, also with a lifespan approach.

Lastly, in clinics, the performance of subjects is normally compared with normality ranges which highly depends on the age ranges that have been considered for the normality definition. Our approach and results may be adopted in clinical practice to assess whether individual balance performance in static and dynamic conditions is in line with the average performance of age-matched people. Indeed, our choice to use a robust fitting method to reduce the effects of outliers (41) allowed us to focus on the average performance of subjects, and not on individual subjects. However, the fitting we are proposing may be used to detect anomalous performance and highlight the early appearance of motor impairments.

To conclude, we highlight a twofold reason why this study could be useful in the clinical environment:

1. It provides a framework—set up and protocol—to assess, in a well-controlled and repeatable manner, balance control in presence of different perturbations as the instability, the predictable, and the unpredictable motion of the surface where one stands.
2. It provides a mathematical description of the decline with age of balance abilities under static and dynamic conditions, providing data from a large population and covering the entire adult lifespan. This approach can be used to evaluate the possible onset of balance problems, separating them from a normal decay of the balance abilities due to age. In fact, subjects who can be considered outliers, falling at the margins or outside of the range of variability of the proposed fitting could have a specific balance problem and must be carefully monitored. This could also allow for early detection of specific balance problems and to plan a timely rehabilitative intervention.

## Limitations

We acknowledge that we did not randomize the proposed five testing conditions, and this could potentially bias the presented results. However, subjects underwent a familiarization phase in which they experienced all the exercises to avoid effects due to initial exposure to a specific exercise and to the device. Also, the exercises were different and kept short. Thus, we did not expect or observe the effects of fatigue or of learning. Nevertheless, if the performance in a specific exercise could be biased by the order of the presentation of tests, we could expect the same effects on the entire population since all subjects were tested following the same order of the five exercises.

Also, in this study, we did not include dynamic tests with eyes closed. Knowing when subjects would fall could be another way to probe balance abilities and the relation with age. However, in designing the study, we decided to keep the protocol safe without forcing participants to face difficult and stressful conditions.

All these *a priori* choices allowed us to have a protocol suitable for testing more conditions, each highlighting different aspects of postural control for a comprehensive and exhaustive assessment, lasting around 5 min.

## Data Availability Statement

The datasets generated and/or analyzed for this study are available from the corresponding author on reasonable request.

## Ethics Statement

The studies involving human participants were reviewed and approved by CE DIBRIS: 012/2020 and Comitato Etico Regionale (CER) Liguria, reference number: 169REG2016. The patients/participants provided their written informed consent to participate in this study.

## Author Contributions

GM, ADL, VS, MC, and AC: contributed to the methodology. GM, MC, and AC: contributed to the software, conducted the formal analysis, and contributed to the writing and original draft preparation. AC, ADL, MC, and GM: contributed to the investigation. AC and MC: supervision. All authors contributed to the conceptualization of the study writing, reviewing, and editing. All authors have read and agreed to the published version of the manuscript.

## Funding

This work was supported by Ministry of Science and Technology, Israel (Joint Israel-Italy lab in Biorobotics Artificial somatosensation for humans and humanoids), and GM was supported by the regione Liguria Ph.D scholarship.

## Conflict of Interest

ADL and VS works for Movendo Technology srl, work for Movendo Technology that commercializes the hunova robotic device used in this study. The remaining authors declare that the research was conducted in the absence of any commercial or financial relationships that could be construed as a potential conflict of interest.

## Publisher's Note

All claims expressed in this article are solely those of the authors and do not necessarily represent those of their affiliated organizations, or those of the publisher, the editors and the reviewers. Any product that may be evaluated in this article, or claim that may be made by its manufacturer, is not guaranteed or endorsed by the publisher.
