# Supplementary material for: A Lifespan Approach to Balance in Static and Dynamic Conditions: The Effect of Age on Balance Abilities
Source: Front Neurol. 2022 Feb 21;13:801142. doi: 10.3389/fneur.2022.801142 (PMC8899125; doi:10.3389/fneur.2022.801142)
Supplement: Supplementary file 1 [file Data_Sheet_1.pdf]

Supplementary Material

In these Supplementary Materials, we report all the preliminary analyses we performed to justify some choices we did in our study.

Since we wanted a clinically applicable and safe protocol where, in conditions that do not imply the risk of falling, we can detect changes in postural parameters that would determine that risk, each performed exercise (exception made by test 5) lasted 20 s. This choice was made a priori by clinicians, who based their choice on their clinical experience, as they expected there were not differences on observing 20 or 30 seconds of these exercises. Also, they wanted to keep the overall evaluation as shorter as possible, indeed we proposed an assessment based on a higher number of shorter tests with respect to the current practice.

For completeness, here, we show the results of the fitting, obtained considering both the 20 s and the 30 s for subjects who performed 30 seconds of evaluation (figure SM1).

The fitting curve obtained from the two sets of data (i.e., 20 and 30 seconds) lead to similar fitting curves. As explained in the method, in our second-order polynomial curve, the only parameter that is not fixed is *a,* the second-order coefficient. In figure SM2, we report the *a*-values and the 95% confidence interval we obtained for each fitting curve. In this figure we reported the results of the paired t-test comparing all the parameters computed independently on age range for the two test's windows. There is a significance difference only in the STD AP for the static test with eyes open, that influences also the sway area, meaning that the variability of the AP oscillations is bigger when considering the 20 seconds time window. Since this significance is not evident in the other tests, to meet the desire of clinicians, we decided to perform assessments lasting 20 seconds for a shorter and safer protocol.

Although this could be a sign that longer tests are preferable in static condition, please notice that this would bias our results on age-relate changes depending visual feedback in static balance. In fact, we reported bigger STD AP in the EC than in EO condition. The fact that EO is bigger when considering 20s with respect to 30 s, it means that we tested the less favorable condition (for finding a difference) and we found that significant.

Then, in figure SM3, we justify the choice of merging together the two lateral perturbations. To assess whether the two perturbations were different, we compared the computed parameters with a paired t-test. As expected, no differences were found, as the postural responses do not differ depending on left and right perturbations.

Here, we also report in table SM-I the R^2 to justify our choice to use the quadratic function. Indeed, as explained in the methods section, we wanted to define the dependency of the postural response on age. We tried to keep the model as simple as possible, hence with the lowest number of parameters.

Here, we show the results of different function we tried:

the quadratic function

$$P = a\,y^2 + b\,y + c \quad \text{with c = 0;}$$

the exponential function

$$P = d\,e^{y} + h \quad \text{with h = 0;}$$

and a power law

$$P = i\,y^{l} + j \quad \text{with j = 0.}$$

Despite for some parameters the quadratic and the power low have similar R^2, we decided to use the quadratic function as it depends on just one parameter.

Lastly, to confirm the strength and validity of our results, we computed the power analysis related to the comparison of performance between subjects under 50 and over 50. Given the effect size, the sample size and α (set at a family-wise error rate of 0,05), we computed the power of our results. Results are shown in table SM-II.

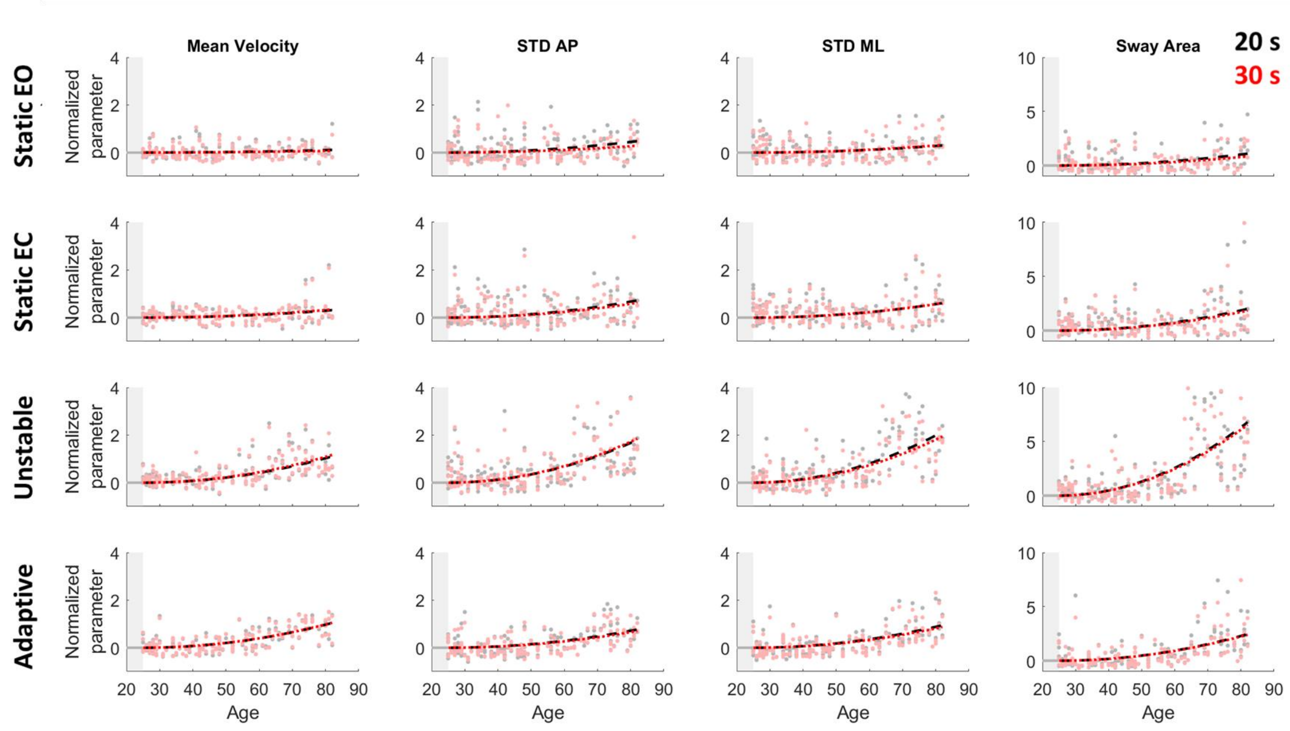

Supplementary Figure 1: Parameters for test 1 to 4 considering 20 s (grey dots for single subjects' data, and black line for the fitting curve) and 30 s (light red dots for single subjects' data, and red line for the fitting curve). Each graph represents how a single parameter changes with age (x-axis: age (years), y-axes: normalized performance indexes). Each row is relative to a different test: namely (from top to bottom) static EO, static EC, unstable, and adaptive. Each column is relative to a specific computed parameter: namely (from left to right) MV, STD AP, STD ML, SA. The colored shaded patch highlights the reference age windows used for normalization (age between 20 and 24 years).

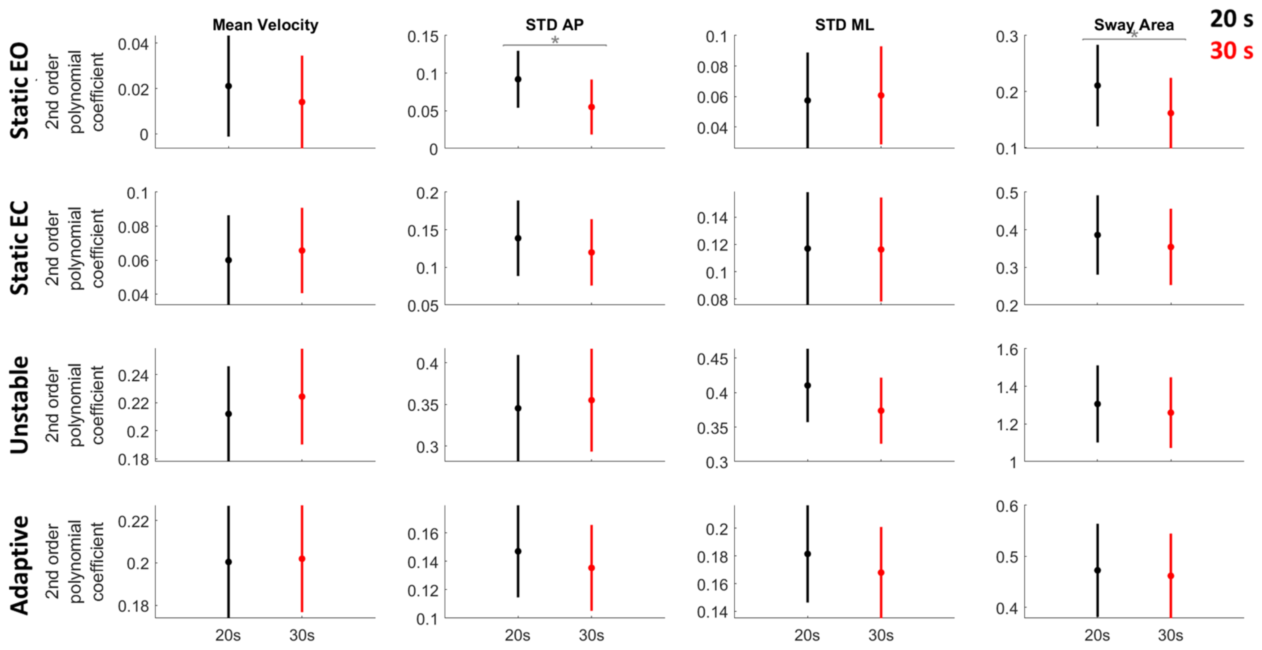

Supplementary Figure 2: Values of the second order coefficient of the fitting curves (A-value) for the 20s (black dot) and the 30s (red dot) test together with the relative 95% confidence intervals (black and red lines). Each row is relative to a different test: namely (from top to bottom) static EO, static EC, unstable, and adaptive. Each column is relative to a specific parameter: namely (from left to right) MV, STD AP, STD ML, SA.

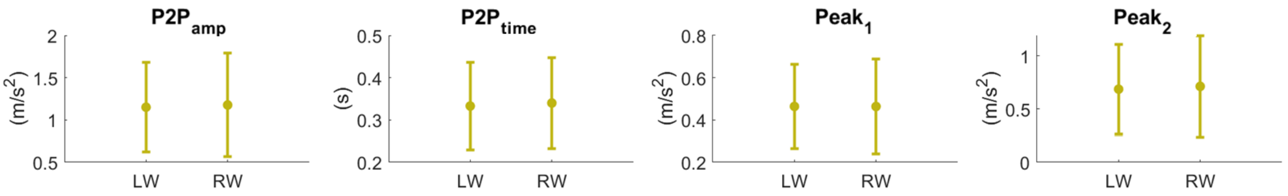

Supplementary Figure 3: Mean and standard deviation of the computed parameters for the two lateral perturbations (LW – leftward and RW – rightward). Each column is relative to a specific parameter: namely the amplitude difference between $Peak_1$ and $Peak_2$ ($P2P_{amp}$), the time difference between $Peak_1$ and $Peak_2$ ($P2P_{time}$), the amplitude of the first peak ($Peak_1$), the amplitude of the rebound ($Peak_2$). Data are shown prior to the normalization. Postural responses do not differ depending on left and right perturbations.

R^2	Parabolic fitting	Power law fitting	Exponential fitting
	Mean Velocity		
Static EO	0,161	0,126	0,112
Static EC	0,090	0,049	0,044
Unstable	0,328	0,332	0,277
Adaptive	0,393	0,379	0,325
	STD AP		
Static EO	0,039	0,034	0,012
Static EC	0,125	0,104	0,097
Unstable	0,393	0,407	0,309
Adaptive	0,358	0,251	0,168
	STD ML		
Static EO	0,110	0,092	0,087
Static EC	0,110	0,043	0,036
Unstable	0,445	0,439	0,413
Adaptive	0,350	0,370	0,307
	Sway Area		
Static EO	0,265	0,235	0,208
Static EC	0,203	0,204	0,185
Unstable	0,616	0,663	0,556
Adaptive	0,456	0,549	0,402

Table SM-I: R^2 for the proposed fitting functions

	MV (m/s)	STD AP(m/s^2)	STD ML(m/s^2)	Area(m^2/s^4)
**Static EO**	16,6%	98,4%	83,1%	95,3%
**Static EC**	42.9%	96,3%	24,7%	74,0%
**Unstable**	100%	100%	100%	100%
**Adaptive**	100%	100%	100%	100%
	**$P2P_{time}$ (s)**	**$P2P_{amp}$ (m/s^2)**	**$Peak_1$ (m/s^2)**	**$Peak_2$ (m/s^2)**
Reactive, FWD	93,5%	100%	100%	99,9%
Reactive, lateral	99,9%	100%	100%	100%

Table SM-II: Post-hoc statistical power of our results, given the effect size, the sample size and α (set at a family-wise error rate of 0,05).
